# The Control of the Feeble-Minded

**Published:** 1921-03-05

**Authors:** 


### THE EDITOR'S LETTER-BOX.

*Correspondence on all subjects is invited, but we cannot in any way be responsible for the opinions expressed by our correspondents, who must give their name and address as a guarantee of good faith, but not necessarily for publication. Correspondents are reminded that brevity of style and conciseness of statement greatly facilitate early insertion.*

### The Control of the Feeble-Minded.

*To the Editor of* The Hospital.

Sir,—While the State is interfering in almost every department of life, until people feel as if they had indeed reached their second childhood and were continually under authority, there is one serious matter where the law seems ever less and less likely to be strengthened. So cautious are our authorities lest they should unjustly call a man insane that they permit almost complete freedom to persons who are certainly far from normal. Only recently a man was sent to prison for a year because he had stabbed another. This was his third similar offence. In one case he had attacked a woman with a saw, and in the second struck a man with a chisel. The second victim died, but the assailant got off on the plea of "justifiable homicide." The prison doctor said that the criminal was "mentally childish," but refused to pronounce him insane. From the point of view of the average citizen, a "mentally childish" man with homicidal tendencies is quite insane enough to be put under permanent preventive control. There is no guarantee that a year's imprisonment will cure these tendencies, but at the end of the year he will walk out free from any guardianship. Surely such weakness should be controlled for its own sake and that of others. In another case, a newspaper tells the tale of a feeble-minded woman, pregnant, who entered the workhouse with her seven children. Her husband also was *non compos mentis*, and the reporter who records the case seems to think that the man had a grievance in not being allowed to go out of the workhouse to look for work, leaving his wife and children behind him. Rather we think of the grievance to the community that any member of such a family should be at large at all. The possibilities of further parenthood on the part of such a couple should be guarded against, and their existing offspring kept under careful control until they reach maturity. The possibilities of reproduction of such a stock are appalling. The improvement of material conditions may do much to prevent the poverty and degradation which we all deplore. But at the back of it we have to face the problem of heredity. We cannot have the "A1" population of which we have heard so much in recent years if we permit uncontrolled breeding from stocks that are "C 3"—or even lower.—I am, Sir, yours faithfully, F. S.

